# Self-objectification and commentary on physical appearance predict exercise dependence in young Chinese males: a study from a cognitive-behavioral perspective

**DOI:** 10.3389/fpsyg.2025.1681877

**Published:** 2025-10-31

**Authors:** Ling Su, WenChi Zou, RongHai Su, MaoChou Hsu, YiLin Gu, SiYu Chen

**Affiliations:** ^1^School of Management, Guangzhou College of Technology and Business, Foshan, China; ^2^School of Business, Macau University of Science and Technology, Macao SAR, China; ^3^College of Physical Education and Sports, Beijing Normal University, Beijing, China; ^4^Department of Recreation Sports Management, Tajen University, Yanpu, Taiwan Province, China; ^5^Child Development Researcher Institute, Beijing, China

**Keywords:** self-objectification, commentary on physical appearance, social physique anxiety, muscle dysmorphia, exercise dependence

## Abstract

Exercise dependence has emerged as a growing concern in China alongside the rapid expansion of fitness culture, yet little is known about its psychological mechanisms in men. Guided by Cash’s cognitive-behavioral model, this study examined whether self-objectification and appearance-related commentary predict exercise dependence through the sequential mediating roles of social physique anxiety and muscle dysmorphia. Data were collected in a four-wave survey over 20 weeks from 354 gym members across 12 clubs in three major Chinese cities. Participants were well-educated male strength-training enthusiasts, had stable incomes, and reported regular training habits, aged 24–45 years. Structural equation modeling supported the hypothesized model: both self-objectification (*B* = 0.08, *p* < 0.05, 95% CI [0.07, 0.29]) and appearance commentary (*B* = 0.21, *p* < 0.05, 95% CI [0.09, 0.34]) predicted exercise dependence indirectly through social physique anxiety and muscle dysmorphia. Findings highlight that evaluative cognitions and appearance-related commentary exacerbate emotional vulnerabilities, which in turn fuel maladaptive exercise behaviors. The study advances theory by extending the cognitive-behavioral model to Chinese men and underscores the importance of addressing both social feedback and emotional regulation in prevention and intervention efforts.

## 1 Introduction

Exercise dependence (ED) has emerged as a critical issue in the study of maladaptive exercise behaviors due to its rising prevalence and negative consequences for physical and psychological well-being (Zhu et al., 2024; Szabo et al., 2018; Cataldo et al., 2021). It is defined as a maladaptive pattern of excessive exercise characterized by physiological, psychosocial, and cognitive symptoms (Downs et al., 2004), and has been linked to anxiety, depression, impaired social functioning, and reduced quality of life (Hausenblas and Downs, 2002; Li, 2014; Szabo et al., 2018; Paradis et al., 2013). Studies across different populations report varying prevalence rates of exercise dependence, with estimates ranging from 7.7–20.9% in Europe (Rudolph, 2018; Corazza et al., 2019; Lichtenstein et al., 2020) to 9–30% among Chinese university students, among whom males consistently exhibit higher risk than females (Li and Nie, 2015; Ding, 2019; Yang et al., 2022). Studies outside China further indicates that men have a higher risk of ED than women (Costa et al., 2013; Dryer et al., 2016; Pope et al., 2000; Riccobono et al., 2020). In contemporary China, the rapid growth of fitness culture and the influence of online media have intensified appearance-based social comparisons, making body image dissatisfaction a salient risk factor for compulsive exercise (Cash et al., 2002; Cataldo et al., 2021; Guo and Ma, 2022). Yet, despite these trends, empirical research on the mechanisms of ED in Chinese men remains scarce (Badenes-Ribera et al., 2019; Jin et al., 2015; Yang et al., 2005). Addressing this gap is essential for understanding how sociocultural and psychological factors interact to shape maladaptive exercise in this population.

Guided by Cash’s (2012) cognitive-behavioral model of body image, this study conceptualizes self-objectification (SO) and commentary on physical appearance (COPA) as antecedents of ED, with social physique anxiety (SPA) and muscle dysmorphia (MD) operating as sequential mediators. According to the model, historical influences (e.g., personality attributes, internalized cultural ideals) and proximal influences (e.g., situational events, interpersonal feedback) jointly shape how individuals evaluate and regulate their body image, and this body image cognition shaped by external standards will trigger emotional experiences and subsequently lead to body image-related behaviors (Cash, 2012; Frederick and Reynolds, 2021; Lewis-Smith et al., 2019; McCreary and Saucier, 2009). SO, defined as adopting a third-person perspective on one’s body and viewing oneself primarily as an object to be evaluated (Calogero, 2012). In this context, SO reflects a historical cognitive vulnerability, characterized by adopting a third-person perspective on one’s body and evaluating oneself primarily as an object (Calogero, 2012). Individuals with higher SO engage in persistent body monitoring and are more likely to experience anxiety regarding how their bodies are perceived by others (Noll and Fredrickson, 1998; Grieve and Helmick, 2008; Schwartz et al., 2010). This externalized self-focus, intensified by social media exposure and cultural ideals of muscularity, reinforces body dissatisfaction and heightens SPA, the anxiety experienced when individuals believe their physique is being judged by others (Hart et al., 1989). Empirical evidence supports this connection: men with elevated SO tend to report greater SPA, particularly in appearance-comparison contexts (Greenleaf and McGreer, 2006; Melbye et al., 2007; Minutillo et al., 2024; Zhu et al., 2024). Accordingly, it is hypothesized that self-objectification is positively associated with social physique anxiety (H1).

While self-objectification captures a cognitive internalization of external standards, commentary on physical appearance represents a proximal social influence that reinforces these standards. Commentary can include positive, negative, or neutral feedback about one’s appearance (Herbozo and Thompson, 2006a). Research suggests that both positive and negative appearance-based remarks contribute to heightened body monitoring and preoccupation (Bailey and Ricciardelli, 2010; Gillen and Lefkowitz, 2009). Even seemingly complimentary comments may perpetuate reliance on external validation, fostering social physique anxiety as individuals anticipate evaluative scrutiny (Nowell and Ricciardelli, 2008; Tanvir et al., 2025). Within the objectification framework, such commentary encourages individuals to adopt an observer’s perspective on their bodies, thereby increasing anxiety about social evaluation (Calogero, 2004; Meyer et al., 2023). Consistent with this reasoning, it is hypothesized that commentary on physical appearance is positively associated with social physique anxiety (H2).

Social physique anxiety, in turn, serves as an emotional mechanism that translates cognitive and social vulnerabilities into psychopathological outcomes. Elevated SPA exacerbates fear of negative evaluation and deepens preoccupation with perceived physical flaws (Tod and Lavallee, 2010; Tod et al., 2016), fostering the development of muscle dysmorphia—a disorder characterized by the obsessive belief that one’s body is insufficiently muscular (Pope et al., 1997; Olave et al., 2021). Individuals with higher SPA are more likely to experience MD symptoms, such as compulsive resistance training, rigid dietary habits, and body concealment (Brunet Segura-García et al., 2010; Sicilia et al., 2016; Duran and Öz, 2022). Evidence from both clinical and community samples confirms that SPA is a strong predictor of MD severity (Zheng et al., 2021; Mastro et al., 2016; Hollander and Aronowitz, 1999; Mastro et al., 2016). Therefore, it is hypothesized that social physique anxiety is positively associated with muscle dysmorphia (H3).

Muscle dysmorphia and exercise dependence, though distinct constructs, are conceptually intertwined. MD involves distorted body image and emotional distress related to perceived muscular inadequacy, which often manifests behaviorally as compulsive exercise (Segura-García et al., 2010; Specter and Wiss, 2014). From a cognitive-behavioral perspective, MD fosters ED by linking maladaptive cognitions and affective dysregulation to compulsive training behaviors (Murray et al., 2010; Olave et al., 2021). Cognitively, MD involves distorted self-perception, which amplifies body dissatisfaction and reinforces exercise as a compensatory behavior (Numanović et al., 2018; Segura-García et al., 2010). Affectively, negative emotions, such as shame and anxiety about one’s physique, prompt the use of exercise for mood regulation (Murray et al., 2010; Olave et al., 2021). Through these two pathways, exercise becomes a rigid strategy to alleviate distress or avoid negative self-evaluation (Specter and Wiss, 2014). Empirical evidence consistently supports the predictive role of MD in ED (Segura-García et al., 2010; Tod and Edwards, 2015; Corazza et al., 2019; Martenstyn et al., 2021; Lev Arey et al., 2025), particularly among male exercisers (Numanović et al., 2018). In summary, MD acts as a significant and reliable antecedent to ED across diverse populations. Therefore, it is hypothesized that muscle dysmorphia is positively associated with exercise dependence (H4).

Integrating these relationships, a sequential mediation model is proposed in which self-objectification and commentary on physical appearance influence exercise dependence through the serial mediation of social physique anxiety and muscle dysmorphia. This pathway captures the progression from maladaptive cognitions and social influences to emotional dysregulation and ultimately to behavioral dependence. In line with the cognition–emotion–behavior sequence posited by the cognitive-behavioral framework (Cash, 2012), this study posits that social physique anxiety and muscle dysmorphia are key emotional and cognitive mechanisms linking appearance-related concerns to compulsive exercise. Thus, two serial mediation hypotheses are proposed: the relationship between self-objectification and exercise dependence is serially mediated by social physique anxiety and muscle dysmorphia (H5a), and the relationship between commentary on physical appearance and exercise dependence is serially mediated by social physique anxiety and muscle dysmorphia (H5b).

**Figure 1:**
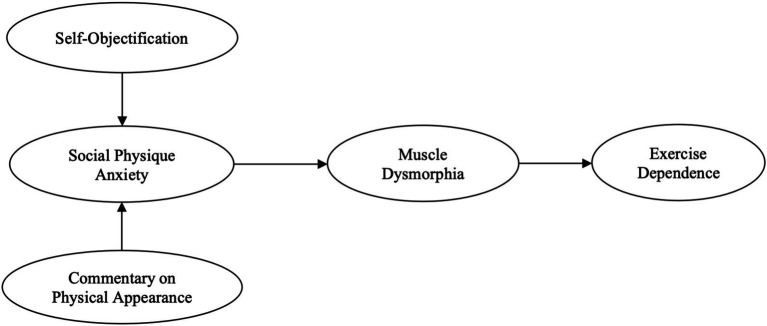
Hypothesized model.

By situating these constructs within a unified cognitive-behavioral framework, this study seeks to clarify how cognitive, social, and emotional processes interact to foster maladaptive exercise behaviors among young Chinese men. The findings are expected to contribute to theoretical refinement of body image research and to inform culturally sensitive interventions for exercise-related psychopathology (Figure 1).

## 2 Materials and methods

### 2.1 Participants and procedure

Data were collected from 12 gym clubs located in Beijing, Shanghai, and Guangzhou. The participants are mainly young and middle-aged male strength fitness enthusiasts who have received a good education and have stable incomes. Most of them have regular training habits. The demographic characteristics of the participants is shown in Table 1. The research team collaborated with customer managers and trainers at these facilities to recruit members willing to participate. To encourage participation, respondents who completed all survey waves received a 100 RMB e-gift voucher (approximately US$15 at the time of data collection).

**Table 1:** Demographic characteristics of participants (*N* = 354).

Characteristic	*n*	%
Age		
24 ~ 30	167	47.18%
31 ~ 35	142	40.11%
36 ~ 45	38	10.73%
≥45	7	1.98%
Educational level		
High school and below	45	12.71%
Undergraduate	199	56.21%
Master’s degree	101	28.53%
Doctor	9	2.54%
Weekly exercise frequency		
1time	118	33.33%
2 ~ 3 times	174	49.15%
4 ~ 5 times	49	13.84%
≥6 times	13	3.67%
Annual income		
<100,000	71	20.06%
100,000 ~ 200,000	87	24.58%
200,000 ~ 300,000	153	43.22%
>300,000	43	12.15%

The annual income is in RMB.

**Figure 2:**
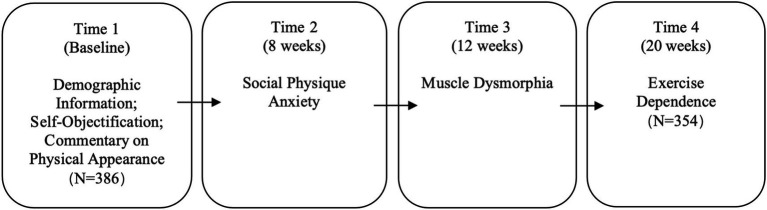
Study flow diagram.

The study employed a four-wave survey design conducted over a 20-week period (study flow presented in Figure 2). All questionnaires were completed in the presence of trained research assistants at the gym clubs. At baseline, 386 male members volunteered and provided informed consent. Participants were assured of confidentiality and informed that they could withdraw at any stage. During the first survey (Time 1), participants reported demographic information and completed measures of self-objectification and commentary on physical appearance. Following Smith et al. (2016), seven professional bodybuilders were excluded at this stage to ensure sample comparability. The second survey (Time 2) was administered 8 weeks later and assessed social physique anxiety. Four weeks after that (12 weeks post-baseline), the third survey (Time 3) measured muscle dysmorphia. The final wave occurred 20 weeks after the initial survey and focused on exercise dependence (Time 4). Across the study, six participants were lost to follow-up and 19 discontinued regular exercise for more than 5 months, rendering them ineligible to complete the survey. The final sample comprised 354 male gym members, aged 24 to 48 years (*M* = 35.28), with an average fat-free mass index (FFMI) of 18.19 (SD = 3.04). Data collection took place between February 3 and June 3, 2024.

### 2.2 Measures

#### 2.2.1 Verbal commentary on physical appearance

The 20 item Verbal Commentary on Physical Appearance Scale (VCOPAS; Herbozo and Thompson, 2006a) was used to assess the frequency and impact of receiving comments from others. VCOPAS includes three subscales: Negative Weight/Shape, Positive Weight/Shape, and Positive General Appearance. This study used only impact ratings. Sample items included “You’ve gained weight” (Negative Weight/Shape subscale), “You are in great shape” (Positive Weight/Shape subscale), and “You are handsome” (Positive General Appearance subscale). Responses were recorded on a five-point Likert scale, ranging from 1 (very negative) to 5 (very positive) (Herbozo et al., 2017b). The VCOPAS has shown good internal reliability and convergent validity in past research (Herbozo et al., 2017a; Herbozo and Thompson, 2006a), and Cronbach’s *α* was 0.90 in this study.

#### 2.2.2 Self objectification

Self-objectification was measured using the Male Assessment of Self-Objectification (MASO; Daniel et al., 2014). The MASO is a 20-item instrument designed to assess the extent to which men define their self-worth based on physical appearance rather than physical competence. Factor analytic results support a two-dimensional structure comprising an appearance-based subscale (13 items; e.g., “sexual appeal,” “well-defined abdominal muscles”) and a competence-based subscale (7 items; e.g., “flexibility”) (Lacey, 2017). The scale produces three scores: appearance-based, competence-based, and a total self-objectification score, calculated by subtracting the mean competence score from the mean appearance score (Lacey, 2017). Higher scores indicate greater self-objectification. Participants rated each attribute on a 5-point Likert scale ranging from 1 (not important at all) to 5 (very important). In the present study, only the total self-objectification score was used for analysis, which demonstrated excellent internal consistency (Cronbach’s *α* = 0.91).

#### 2.2.3 Social physique anxiety

The nine item short form Social Physique Anxiety Scale (SPAS; Martin et al., 2007) was used to measure social physique anxiety. The Social Physique Anxiety Scale measures the anxiety that an individual experiences through interpersonal judgments and evaluations focused on their physiques. One of the sample items was “In the presence of others, I feel apprehensive about my physique/figure.” Responses to this scale used a five-point Likert scale ranging from 1 (not at all) to 5 (extremely). The short form of the SPAS has been shown to be reliable and valid (McCreary and Saucier, 2009), and Cronbach’s α was 0.85 in this study.

#### 2.2.4 Muscle dysmorphia

The 15 item Drive for Muscularity Scale (DMS; McCreary and Sasse, 2000) assesses the males’ attitudes and behaviors associated with achieving muscularity (McCreary et al., 2004). Responses to this scale used a five-point Likert scale ranging from 1 (never) to 5 (always) to examine male’s attitudes toward muscularity. A sample item is “I wish I was more muscular.” Prior research has demonstrated that the Drive for Muscularity Scale has consistently acceptable reliability, as well as good construct, concurrent, convergent, and discriminant validity (McCreary and Saucier, 2009). Cronbach’s *α* of the DMS in this study was 0.87.

#### 2.2.5 Exercise dependence

The Exercise Dependence Scale (EDS; Downs et al., 2004) used in this study is a multidimensional questionnaire comprising 21 items across seven dimensions: tolerance, withdrawal, intention effects, lack of control, time, continuance, and reduction in other activities. Sample items include “I continually increase my exercise intensity to achieve the desired effects or benefits” (tolerance subscale) and “I exercise to avoid feeling anxious” (withdrawal subscale). Participants responded on a 6-point Likert scale ranging from 1 (never) to 6 (always), based on the DSM-IV criteria for substance dependence. Previous studies have frequently calculated subscale scores independently when assessing exercise dependence. Individuals are often classified as being at high risk when they obtain scores of 15 or higher on at least three subscales (Festino et al., 2024; Yang et al., 2023), and higher scores on individual subscales reflect more pronounced dependence symptoms (Zou et al., 2022). This scoring practice suggests that the EDS reflects a formative conceptualization of exercise dependence, in which the construct is composed of its seven behavioral dimensions rather than reflected by them (Bollen and Bauldry, 2011; Bollen and Diamantopoulos, 2017). Following this causal-formative measurement framework, each dimension was assumed to contribute equally to the overall construct. Therefore, the overall exercise dependence score was calculated as the mean of the seven subdimension scores, rather than the mean of all 21 items combined, to better reflect the multidimensional structure of the construct. Higher scores indicate a greater tendency toward exercise dependence. The Cronbach’s α coefficient for the EDS in this study was 0.86, demonstrating good internal consistency.

### 2.3 Ethics

This study was approved by the ethics committee of Macau University of Science and Technology (MSB-202410). The questionnaire included statements indicating voluntary participation. All study participants were asked to provide formal consent prior to participation, and were informed that they were free to withdraw at any time without incurring any consequences. The cover page of the questionnaire outlined the study objectives and assured the participants that their anonymity and confidentiality would be strictly maintained. Participants were also assured that their responses would be utilized exclusively in academic research. To ensure confidentiality, completed questionnaires were immediately sealed in envelopes. They were placed in envelopes and coded with a researcher-assigned identification number to facilitate matching across the three data collection waves.

### 2.4 Statistical analysis

All statistical analyses were conducted using SPSS 26.0 for descriptive statistics and reliability testing, and AMOS 22.0 for confirmatory factor analysis (CFA) and structural equation modeling (SEM). First, descriptive statistics (means, standard deviations) and correlations were calculated for all study variables. Internal consistency reliability was examined using Cronbach’s alpha (Cronbach, 1951), with coefficients above 0.70 considered acceptable. Second, CFA were performed to evaluate the distinctiveness of the five latent constructs: self-objectification, commentary on physical appearance, social physique anxiety, muscle dysmorphia, and exercise dependence. Competing measurement models were compared following recommendations by Hu and Bentler (1999) and Kline (2005). Model fit was evaluated using the chi-square statistic (χ^2^), the comparative fit index (CFI), the non-normed fit index (NNFI/TLI), the root mean square error of approximation (RMSEA) and its 90% confidence interval, and the standardized root mean square residual (SRMR). Third, SEM was employed to test the hypothesized paths among the five constructs. The hypothesized model was compared with alternative models, and model comparison was based on changes in χ^2^ (Δχ^2^), along with differences in CFI and RMSEA. Finally, mediation analyses were conducted to test the proposed serial mediation effects of social physique anxiety and muscle dysmorphia between (a) self-objectification and exercise dependence and (b) commentary on physical appearance and exercise dependence. To provide robust estimates of indirect effects, the bootstrapping procedure with 5,000 resamples was used to generate bias-corrected 95% confidence intervals (CI). Indirect effects were considered statistically significant when the CI did not include zero.

## 3 Results

### 3.1 Confirmatory factor analyses

To establish the distinctiveness of the study variables, we conducted confirmatory factor analyses (CFA) and compared the hypothesized measurement model with several plausible alternatives. Consistent with recommended evaluation criteria (Hu and Bentler, 1999; Kline, 2005), the hypothesized five-factor model, comprising self-objectification, commentary on physical appearance, social physique anxiety, muscle dysmorphia, and exercise dependence, demonstrated a superior fit relative to competing models (see Table 1). These results confirm the discriminant validity of the five constructs (Table 2).

**Table 2:** Confirmatory factor analysis of measures.

Model	Factors	χ2	df	RMSEA	CFI	SRMR
Baseline model	Five factors	276.87	125	0.06	0.92	0.03
Alternative Mode 1	Four factors	432.19	129	0.09	0.83	0.06
Alternative Model 2	Four factors	425.02	129	0.09	0.82	0.06
Alternative Model 3	Four factors	367.77	129	0.07	0.85	0.05
Alternative Model 4	Three factors	544.18	132	0.11	0.71	0.08
Alternative Model 5	Three factors	569.25	132	0.11	0.76	0.09
Alternative Model 6	Three factors	718.34	132	0.13	0.60	0.12
Alternative Model 7	One factor	971.81	135	0.20	0.48	0.14

Model 1: Self-objectificationandcommentaryonphysicalappearancewerecombinedintoone factor. Model 2: Socialphysiqueanxietyandmuscledysmorphiawerecombinedintoonefactor. Model 3: Muscle dysmorphia and exercise dependence were combined into one factor. Model 4: Self-objectification and commentary on physical appearance were combined in to one factor. Social physique anxiety and muscle dysmorphia combined in to one factor. Model 5: Social physique anxiety and muscle dysmorphia were combined in to one factor; Self objectification and exercise dependence were combined in to one factor. Model 6: Social physique anxiety and muscle dysmorphia were combined in to one factor; commentary on physical appearance and exercise dependence were combined in to one factor. Model 7: All five variables were combined in to one factor. *p < 0.05; **p < 0.01.

### 3.2 Descriptive statistics and correlations

Descriptive statistics and correlations among all study variables are presented in Table 3. The first four variables were measured on a 5-point scale, while exercise dependence was measured on a 6-point scale. Overall, participants reported moderately high levels of SO (*M* = 4.27) and ED (*M* = 4.43), indicating strong appearance awareness and a tendency toward compulsive exercise among Chinese male strength-training enthusiasts. The mean scores for COPA (*M* = 3.53), SPA (*M* = 3.44), and MD (*M* = 3.89) also reflected noticeable body-related concern and dissatisfaction.

**Table 3:** Descriptive statistics and correlations.

Variables	M	SD	1	2	3	4	5
1. Self-objectification	4.27	1.13	—				
2. Commentary on physical appearance	3.53	0.98	0.46^**^	—			
3. Social physique anxiety	3.44	0.95	0.38^**^	0.42^**^	—		
4. Muscle dysmorphia	3.89	0.99	0.29^**^	0.37^**^	0.45^**^	—	
5. Exercise dependence	4.43	0.91	0.40^**^	0.48^**^	0.39^**^	0.51^**^	—

**p < 0.01.

Correlation analyses revealed that both SO and COPA were significantly and positively associated with SPA, which in turn correlated positively with MD and ED (*p* < 0.01). These findings provide preliminary support for the proposed cognitive-emotional pathway linking appearance-related cognitions and emotions to maladaptive exercise behavior.

### 3.3 Structure equation modeling

**Figure 3:**
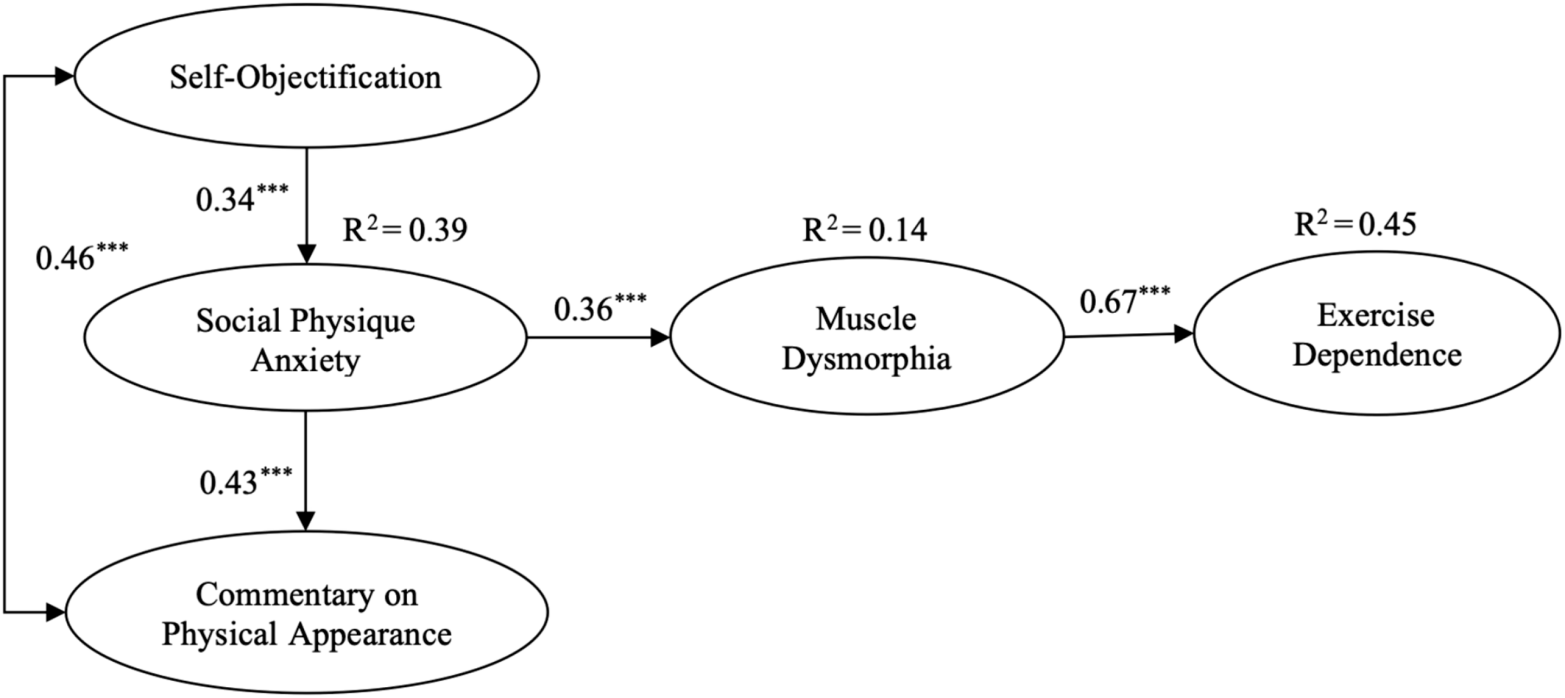
Standardized direct effects and percentage of variance explained in the hypothesized structural model (^***^p < 0.001).

The hypothesized structural model was tested using AMOS 22. As shown in Figure 3, the model provided a good fit to the data: χ^2^ (516) = 1025.39, *p* < 0.001, RMSEA = 0.08 (90% confidence interval CI [0.08, 0.10]), CFI = 0.94, NNFI = 0.93, and SRMR = 0.04. To test the fit of a more fully saturated model, two additional paths were added to the hypothesized model, namely, one path each from self-objectification and commentary on physical appearance to exercise dependence. This model provided a good fit for the data, χ^2^(163) = 479.75 (*p* < 0.001), RMSEA = 0.05 (90% confidence interval CI [0.11, 0.39]), CFI = 0.94, NNFI = 0.94, and SRMR = 0.03. However, it did not provide a significantly better fit than the hypothesized model [Δχ^2^(2) = 8.92, *p* < 0.05].

### 3.4 Mediation analyses

To test the proposed serial mediation hypotheses, bootstrapping analyses with 5,000 resamples were conducted. The results are summarized in Table 4. For SO, the direct effect on ED was significant (*B* = 0.35, SE = 0.13, *p* < 0.01, 95% CI [0.10, 0.63]), indicating that higher levels of self-objectification directly predicted stronger exercise dependence. In addition, SO exerted a significant indirect effect on ED through SPA alone (*B* = 0.09, SE = 0.05, *p* < 0.05, 95% CI [0.01, 0.24]) and a serial indirect effect through both SPA and MD (*B* = 0.08, SE = 0.05, *p* < 0.05, 95% CI [0.07, 0.29]). The total effect of SO on ED remained significant (*B* = 0.52, SE = 0.21, *p* < 0.01, 95% CI [0.12, 0.97]), suggesting both direct and mediated pathways contribute to the overall relationship.

**Table 4:** Path effect of self-objectification and commentary on physical appearance on exercise dependence.

IV	Direct effects		Indirect effects				Total effects	
	B (SE)	95% CI	Via SPA		Via SPA and MD		B (SE)	95% CI
			B (SE)	95% CI	B (SE)	95% CI		
SO	0.35 (0.13)^**^	[0.10, 0.63]	0.09 (0.05)^*^	[0.01, 0.24]	0.08 (0.05)^*^	[0.07, 0.29]	0.52 (0.21)^**^	[0.12, 0.97]
COPA	0.62 (0.10)^***^	[0.36, 0.85]	0.15 (0.08)^*^	[0.03, 0.32]	0.21 (0.07)^*^	[0.09, 0.34]	0.98 (0.24)^***^	[0.46, 1.33]

Bootstrap = 5,000; CI = confidence interval; IV = Independent Variables; SO = Self-objectification; COPA = Commentary on Physical Appearance; SPA = Social Physique Anxiety; MD = Muscle Dysmorphia; ED = Exercise Dependence. ^*^p < 0.05; ^**^p < 0.01; ^***^p < 0.001.

Similarly, for COPA, the direct effect on ED was strong and significant (*B* = 0.62, SE = 0.10, *p* < 0.001, 95% CI [0.36, 0.85]). COPA also showed a significant indirect effect via SOPA alone (*B* = 0.15, SE = 0.08, *p* < 0.05, 95% CI [0.03, 0.32]) and a serial indirect effect via SOPA and MD (*B* = 0.21, SE = 0.07, *p* < 0.05, 95% CI [0.09, 0.34]). The total effect of COPA on ED was likewise significant (*B* = 0.98, SE = 0.24, *p* < 0.001, 95% CI [0.46, 1.33]).

Taken together, these findings provide robust support for the hypothesized serial mediation model, confirming that both SO and COPA influence ED through the sequential emotional mechanisms of SPA and MD. This pattern highlights the central role of body-related emotional experiences in translating cognitive and social evaluations into maladaptive exercise behaviors.

## 4 Discussion

The present study concentrated on Chinese men, as prior research within China has repeatedly suggested that men may be at greater risk of developing exercise dependence and therefore warrant particular attention (Liao et al., 2010; Yang et al., 2022). The findings verified that self-objectification and commentary on physical appearance, as cognitive factors, elicited social physique anxiety and muscle dysmorphia, which in turn intensified exercise dependence. This male-focused mechanism resonates with evidence from Laynes et al. (2022), who also investigated male participants and confirmed the role of body dissatisfaction and anxiety in driving exercise dependence.

However, not all studies point to the same gendered pattern (Modolo et al., 2011; Festino et al., 2024). Especially, Festino et al. (2024) reported that female participants, particularly volleyball players, exhibited both higher body dissatisfaction and higher exercise dependence scores compared to their male counterparts. Drawing on objectification theory (Calogero, 2004), the authors suggested that women’s greater vulnerability to body-related concerns may place them at heightened risk for maladaptive exercise. This contrasts with the present findings, which emphasized male vulnerability in the Chinese cultural context. Together, these results highlight that body dissatisfaction can serve as a common mechanism underlying exercise dependence, but the degree to which it operates in men versus women appears to vary across samples and cultural environments. Importantly, Griffiths et al. (2015) cautioned that gender comparisons of exercise dependence may be problematic due to the lack of scalar measurement invariance in the Exercise Addiction Inventory. This raises the possibility that some previously reported gender differences may reflect methodological artifacts rather than genuine disparities, and thus conclusions about which gender is “more at risk” must be approached cautiously.

Furthermore, cross-cultural factors offer another perspective to explain the relationship between gender and sports dependence. Zandonai et al. (2021), for instance, compared Italian and Japanese runners and found exercise dependence prevalence rates of 4.4 and 0%, respectively, which might suggest the potential influence of “national culture” in shaping physical ideals and exercise behaviors. Therefore, the differences between the research results of this study in China and those of Festino et al. (2024) in Italy may also support the explanation of this perspective. Cultural background may influence which gender is more vulnerable.

In sum, the present study demonstrated that cognitive factors such as self-objectification and commentary on physique appearance elicited emotional experiences of social physique anxiety and muscle dysmorphia, which subsequently reinforced exercise dependence in Chinese men. This pathway highlights how evaluative cognitions about the body can generate maladaptive affective responses, ultimately driving excessive exercise. However, recent findings suggest that it may not be dissatisfaction itself, but rather the way individuals respond to these negative evaluations, that most strongly contributes to exercise dependence.

Zou et al. (2022) reported that body image inflexibility, that the rigid and avoidant response to negative body-related thoughts and emotions, was a significant predictor of exercise dependence, whereas simple indices of body dissatisfaction and BMI were not. These results indicate that the vulnerability to exercise dependence is shaped not only by what individuals think about their bodies, but also by their capacity to regulate these cognitions flexibly. Importantly, Zou et al. also found generalized anxiety to be a significant predictor of exercise dependence, underscoring the broader role of emotional dysregulation in the development of maladaptive exercise.

Taken together, the findings of the present study and Zou et al. (2022) may be viewed as complementary rather than contradictory. Whereas the present study identified the specific content of cognitive-emotional processes (self-objectification → physique-related anxiety/dysmorphia), Zou et al. (2022) highlighted body image inflexibility, a subjective inability to regulate negative body-related experiences. The key distinction, therefore, is this study focused on universal cognitive-emotional mechanisms, while Zou et al. (2022) emphasized individual differences in emotional flexibility. This difference helps explain the complexity of exercise dependence: it arises both from objective mechanisms common to most people and from subjective variation in how effectively individuals manage these experiences (Hayes et al., 2006; Sandoz et al., 2019; Alcaraz-Ibáñez et al., 2018; Miller et al., 2023). Therefore, future research could pay more attention to those individual psychological variables that affect exercise addiction and can be improved, and it is more likely to propose effective strategies for preventing and alleviating exercise addiction based on these studies rather than merely focusing on universal mechanisms.

### 4.1 Theoretical implications

This study makes several contributions. First, it extends Cash’s (2012) cognitive-behavioral model by showing how self-objectification and appearance-related commentary trigger social physique anxiety and muscle dysmorphia, ultimately leading to exercise dependence. By testing this pathway in a Chinese male sample, the study highlights how contextual factors shape body image perception, emotional experiences, and behavioral symptoms. These findings add empirical support to cognitive-behavioral explanations of body image disturbances and maladaptive exercise, especially in non-Western cultural contexts.

Second, the results contribute to the cross-cultural literature on masculinity and body image. Previous studies have emphasized the role of sociocultural ideals in shaping male body concerns in both Western and Eastern contexts. This study adds to that line of work by showing how the pursuit of muscularity among Chinese men is increasingly tied to social commentary and self-objectification, reflecting a cultural shift toward valuing strength and muscular appearance.

Finally, by integrating emotional mechanisms with emerging evidence on psychological flexibility (Zou et al., 2022), the study underscores that exercise dependence is driven not only by universal cognitive-emotional pathways but also by individual differences in regulatory capacity. This suggests that maladaptive exercise behaviors cannot be fully explained without considering both shared psychological mechanisms and variability in how people manage negative body-related experiences.

### 4.2 Practical implication

The findings provide practical insights for prevention and intervention. Clinicians should assess not only the presence of body image concerns but also the role of appearance-related comments and the emotional responses they evoke. Since social physique anxiety and muscle dysmorphia can intensify exercise dependence, therapy should focus on helping clients recognize and challenge maladaptive beliefs, reframe negative commentary, and strengthen self-esteem. Cognitive Behavioral Therapy techniques such as cognitive restructuring and reframing have been shown to reduce the impact of self-objectification and external evaluations (Grieve et al., 2009).

Beyond cognitive restructuring, flexibility-based approaches should be incorporated. Training men to accept body-related thoughts and emotions without rigid avoidance may buffer the pathway to dependence. Psychological flexibility can be fostered through mindfulness and acceptance-based interventions (Hayes et al., 2006; Sandoz et al., 2019). Preventive programs targeting adolescents are particularly important, as early exposure to negative appearance-related commentary may shape long-term body dissatisfaction and maladaptive exercise behaviors.

### 4.3 Limitations

This study has limitations that point to avenues for further research. First, the cross-sectional design limits causal inference. Longitudinal and experimental studies are needed to clarify whether social physique anxiety and muscle dysmorphia are antecedents or consequences of exercise dependence. Second, the study did not account for environmental factors, such as whether exercising in public settings moderates the link between physique anxiety and dependence. Future research should test how situational contexts influence these relationships. Third, appearance-related commentary was treated broadly. Prior work suggests that comments from specific sources (e.g., parents, peers, romantic partners) have different effects (Schuster et al., 2013). Future studies should disentangle how the source, timing, and content of comments influence men’s body image and exercise behaviors. Finally, research should move beyond universal cognitive-emotional pathways to focus more on individual differences in psychological flexibility. Examining how flexibility interacts with cultural norms and personal vulnerabilities could provide a more nuanced understanding of exercise dependence and inform more tailored intervention strategies.

## Data Availability

The raw data supporting the conclusions of this article will be made available by the authors, without undue reservation.
